# A YWHAZ Variant Associated With Cardiofaciocutaneous Syndrome Activates the RAF-ERK Pathway

**DOI:** 10.3389/fphys.2019.00388

**Published:** 2019-04-08

**Authors:** Ivan K. Popov, Susan M. Hiatt, Sandra Whalen, Boris Keren, Claudia Ruivenkamp, Arie van Haeringen, Mei-Jan Chen, Gregory M. Cooper, Bruce R. Korf, Chenbei Chang

**Affiliations:** ^1^Department of Cell, Developmental and Integrative Biology, The University of Alabama at Birmingham, Birmingham, AL, United States; ^2^HudsonAlpha Institute for Biotechnology, Huntsville, AL, United States; ^3^UF de Génétique Clinique, Hôpital Armand Trousseau, Assistance Publique Hôpitaux de Paris, Centre de Référence Maladies Rares des Anomalies du Développement et Syndromes Malformatifs, Paris, France; ^4^Department of Clinical Genetics, Leiden University Medical Center, Leiden, Netherlands; ^5^Department of Genetics, The University of Alabama at Birmingham, Birmingham, AL, United States

**Keywords:** YWHAZ, CFC, RASopathy, Raf, Erk activation, *Xenopus*

## Abstract

Cardiofaciocutaneous (CFC) syndrome is a genetic disorder characterized by distinctive facial features, congenital heart defects, and skin abnormalities. Several germline gain-of-function mutations in the RAS/RAF/MEK/ERK pathway are associated with the disease, including *KRAS, BRAF, MEK1*, and *MEK2*. CFC syndrome thus belongs to a group of disorders known as RASopathies, which are all caused by pathogenic mutations in various genes encoding components of the RAS pathway. We recently identified novel variants in *YWHAZ*, a 14-3-3 family member, in individuals with a phenotype consistent with CFC that may potentially be deleterious and disease-causing. In the current study, we take advantage of the vertebrate model *Xenopus laevis* to analyze the functional consequence of a particular *YWHAZ* variant, S230W, and investigate the molecular mechanisms underlying its activity. We show that compared with wild type *YWHAZ*, the S230W variant induces severe embryonic defects when ectopically expressed in early *Xenopus* embryos. The S230W variant also rescues the defects induced by a dominant negative FGF receptor more efficiently and enhances Raf-stimulated Erk phosphorylation to a higher level than wild type *YWHAZ*. Although neither *YWHAZ* nor the variant promotes membrane recruitment of Raf proteins, the variant binds to more Raf and escapes phosphorylation by casein kinase 1a. Our data provide strong support to the hypothesis that the S230W variant of *YWHAZ* is a gain-of-function mutation in the RAS-ERK pathway and may underlie a CFC phenotype.

## Introduction

Cardiofaciocutaneous (CFC) syndrome is an autosomal dominant genetic disorder characterized by distinctive facial features, heart malformation, and skin abnormalities (Roberts et al., 2006). Affected individuals typically have a prominent forehead with bitemporal constriction, hypoplastic supraorbital ridges, orbital hypertelorism, down-slanting palpebral fissures, and a depressed nasal bridge. Heart defects include pulmonic stenosis, atrial septal defect, and hypertrophic cardiomyopathy. Most patients have dry, hyperkeratotic, scaly skin and sparse and curly hair. Many of these clinical manifestations overlap with those of Noonan syndrome or Costello syndrome. These phenotypically overlapping syndromes are collectively referred to as RASopathies, owing to the fact that they all share germline gain-of-function mutations in genes encoding components of the RAS-RAF-MEK-ERK pathway (Aoki et al., 2008; Simanshu et al., 2017; Bustelo et al., 2018; Dard et al., 2018).

RAS signaling transmits external cues, such as growth factors, cytokines, and extracellular matrix factors, to intracellular machineries to govern cell proliferation, differentiation, and survival (Simanshu et al., 2017). At the core of the RAS signaling pathway are the RAS family of small GTPases and the kinase cascade comprising RAF, MEK and ERK kinases and the KSR scaffolding protein (Lavoie and Therrien, 2015; Simanshu et al., 2017). Pathway activation is achieved through binding of membrane receptors to their ligands, which stimulates RAS guanosine nucleotide exchange factors (GEFs), such as SOS, that promote the GTP-bound, active form of RAS. Membrane-associated RAS-GTP then recruits RAF family kinases to the plasma membrane, facilitating their dimerization and activation (Weber et al., 2001; Light et al., 2002). Active RAF interacts with KSR scaffold proteins to recruit and activate MEK, leading to ERK phosphorylation (Therrien et al., 1996; Muller et al., 2001; Roy et al., 2002; Brennan et al., 2011; Lavoie et al., 2018). This in turn allows ERK to phosphorylate various effector proteins to influence cell cycle, apoptosis, and differentiation (Khokhlatchev et al., 1998; Casar et al., 2008). Germline mutations in genes encoding different components or regulators of the RAS pathway have been shown to be responsible for the congenital anomalies displayed in individuals with RASopathies. For example, about 75% of CFC patients have gain-of-function (GOF) variants in the *B-RAF* gene, and GOF variants in *K-RAS, MEK1* and *MEK2* are also found in some affected individuals (Aoki et al., 2008; Bustelo et al., 2018; Dard et al., 2018). Noonan syndrome is associated with variants in the gene encoding tyrosine phosphatase SHP2 (*PTPN11*, 50% of patients), an activator of the RAS signaling, as well as in genes of *SOS1, C-RAF*, and other pathway components (Bustelo et al., 2018; Dard et al., 2018). Costello syndrome seems to arise mainly from mutations in *H-RAS*, with more than 90% of affected individuals having pathogenic variants in the gene and a minority harboring mutations in other genes, such as *K-RAS, B-RAF*, and *MEK1* (Bustelo et al., 2018; Dard et al., 2018). Variants at different sites in the same gene can be found with distinct frequencies in affected individuals, and both kinase-activating and kinase-impairing variants, especially in CFC-associated *B-RAF*, can lead to ERK phosphorylation and pathway activation (Garnett et al., 2005; Aoki et al., 2008; Anastasaki et al., 2009; Heidorn et al., 2010; Freeman et al., 2013). This highlights disease heterogeneity and the complexity of RASopathies, and implies that other regulators of RAS signaling may modulate disease phenotypes.

A key intermediary in the RAS pathway that has not been associated with the RASopathies previously is the 14-3-3 family of proteins (Aitken, 2006; Morrison, 2008; Sluchanko, 2018). The 14-3-3 family contains seven members in mammals, which are 14-3-3β, γ, 𝜀, σ, ζ, τ and η, also known as *YWHAB, YWHAG, YWHAE, SFN, YWHAZ, YWHAQ* and *YWHAH* in humans. 14-3-3 proteins form homo- or heterodimers amongst themselves and are conserved regulators of myriad signals via their binding to hundreds of partner proteins, most of which contain consensus motifs surrounding phosphorylated serine or threonine residues. Binding of 14-3-3 proteins can lead to conformational changes of their partners, masking of sequence or structural features in the partners, and/or facilitation of protein interaction and complex formation of the partners (Aitken, 2006; Morrison, 2008; Obsil and Obsilova, 2011). One of the earliest identified and best characterized interaction partners of 14-3-3 are RAF family members (Fantl et al., 1994). 14-3-3 proteins bind to two conserved phosphoserine residues in RAF: one in the N-terminal regulatory region and the other near the C-terminal end of the RAF kinase domain (Muslin et al., 1996; Rommel et al., 1996; MacNicol et al., 2000; Hekman et al., 2004; Fischer et al., 2009). 14-3-3 dimers can either bind phosphoserine residues on the same molecule to hold RAF proteins in an inactive conformation or bridge two molecules by binding the *C-terminal* phosphoserine on each, thereby stabilizing active RAF dimers (Tzivion et al., 1998). Activation of RAF by RAS results in release of 14-3-3 binding from the N-terminal phosphoserine residue, but does not affect 14-3-3 association with the C-terminal residue (Rommel et al., 1996). These observations lead to the current model that in the absence of stimulating signals, 14-3-3 binds to cytosolic RAF at both the *N*- and the *C-terminal* sites to lock the protein in a closed conformation to prevent basal signaling. Upon RAS activation, RAF is recruited to the plasma membrane via its *N-terminal* domain and dissociates from 14-3-3 at its *N-terminal* site. However, 14-3-3 still binds to RAF at the *C-terminal* site, and this binding is required for RAF activation, possibly by facilitating RAF dimerization and/or formation of a protein complex with other factors, such as KSR (Aitken, 2006; Freeman et al., 2013). Hence, depending on the presence or absence of activating signals, 14-3-3 can switch from an inhibitor to an activator of the RAS/RAF pathway.

We have identified several *YWHAZ* variants in individuals with neurodevelopmental phenotypes, two of which had a clinical diagnosis of a RASopathy. However, the consequence of these variants on 14-3-3 function has not been described, and thus their pathogenicity is unclear. In this study, we employed the *Xenopus* model system to characterize the functional effect of *YWHAZ* variation, seeking to dissect molecular mechanisms underlying the activity of the S230W variant. We report that the S230W variant induced more severe embryonic defects than wild type *YWHAZ* when expressed in early *Xenopus* embryos. Though the variant did not enhance membrane recruitment of Raf, it bound to more Raf and activated Erk phosphorylation to a higher level than the wild type protein. Our data indicate that the S230W variant promotes Raf/Erk signal transduction and likely causes gain-of-function in RAS signaling in humans, supporting the hypothesis that the *YWHAZ* variant is a new RASopathy-associated allele.

## Results

### Identification of YWHAZ Variants in Human Patients

**FIGURE 1:**
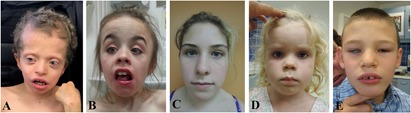
Facial features of probands with variations in *YWHAZ*. **(A)** Proband 1. **(B)** Proband 2. **(C)** Proband 3 (photo taken at the age of 13 and a half years old). **(D)** Proband 4 (photo taken at the age of 4 years and 8 months old). **(E)** Proband 5 (photo taken at the age of 7 years and 5 months old). Written informed consent to publish pictures was obtained from all families.

Through a genome sequencing research study, we identified a heterozygous genetic variant in *YWHAZ* (c.689C > G, p.S230W) in a male with a clinical diagnosis of CFC syndrome (proband 1, Figure 1A, Supplementary Text 1). He has short stature, global developmental delay, bilateral proptosis and ptosis, high forehead, pulmonic stenosis, hyperkeratosis, and seizures. Previous clinical testing included a negative RASopathy panel, but trio genome sequencing revealed a *de novo* serine to tryptophan change in *YWHAZ* at amino acid (aa) position 230, a location close to the *C-terminal* end and two residues away from a known casein kinase phosphorylation site at T232 (Supplementary Table 1). This missense variant is extremely rare and is absent from the gnomAD population database (Lek et al., 2016). The alteration is predicted to have a deleterious effect by multiple lines of computational evidence (SIFT score of 0; GERP score of 4.60; CADDv1.4 score of 33), though its specific functional consequences have not been examined. Through the data-sharing platform GeneMatcher (Sobreira et al., 2015), another *YWHAZ* variant was found in a child who also had a clinical diagnosis of RASopathy (proband 2, Figure 1B, Supplementary Text 1). The female individual has short stature, low body weight, and facial features similar to other patients with RASopathies, including a triangular face and a mild ptosis (Figure 1B, Supplementary Text 1). Trio exome sequencing identified a heterozygous *de novo YWHAZ* variant (c.157G > A, p.G53R) that results in an amino acid change from glycine to arginine at residue 53 (Supplementary Table 1). This missense variant is also absent from the gnomAD population database (Lek et al., 2016) and has multiple lines of computational evidence predicting a deleterious effect (SIFT score of 0; GERP score of 5.64; CADDv1.4 score of 32). Like S230W, the specific functional impact of this sequence variant has not been examined. Three additional *YWHAZ* variants were also identified in three individuals with distinct phenotypes (Probands 3–5, Figure 1C–E, Supplementary Table 1, Supplementary Text 1). While one of these probands harbors a missense variant (S145L, proband 3, Figure 1C), two others have premature termination (E14X, proband 4, Figure 1D) and frameshift (S230Yfs^∗^44, proband 5, Figure 1E) variants, respectively. Each of these probands shows developmental delay or intellectual disability, but none has cardiac or skin abnormalities (Supplementary Table 1, Supplementary Text 1). The distinct clinical features of individuals with different *YWHAZ* variants suggest that the altered gene products may affect the protein function differently. While premature termination of *YWHAZ* most likely represents a loss-of-function allele, the change in activities of the other variants is less clear. The presence of other gene variants in several of the probands (Supplementary Text 1) also makes it difficult to confidently relate clinical phenotypes to *YWHAZ* variation. Because of this, the variants can only be classified as variants of uncertain significance (VUS) according to the ACMG classification guideline (Richards et al., 2015). To explore possible pathogenicity of the *YWHAZ* variants, direct functional characterization is required. In the following, we used the *Xenopus* embryonic system to investigate the functional consequence of the amino acid changes, with a focus specifically on the S230W variant.

### The YWHAZ(S230W) Variant Induces More Severe Embryonic Defects Than Wild Type YWHAZ

**FIGURE 2:**
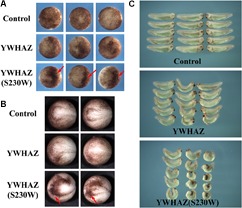
*YWHAZ*(S230W) induces more severe embryonic defects than wild type *YWHAZ*. **(A)** Ectopic expression of *YWHAZ*(S230W) in the animal region induces darkly pigmented cells at the gastrula stages. Arrows point to the darkly pigmented cell clusters. **(B)** Clusters of darkly pigmented cells induced by ectodermal expression of *YWHAZ*(S230W) persist to the neurula stages. Arrows point to the darkly pigmented cell clusters. **(C)** Overexpression of wild type *YWHAZ* leads to defects in the head structure and bent body axis in the resulting tadpoles. Expression of the *YWHAZ*(S230W) variant induces more severe defects than *YWHAZ*, with the tadpoles displaying a significantly shortened and severely bent body axis in addition to stronger defects in the head structure. The experiment has been repeated four times, with 10 to 18 treated embryos in each experiment. In all the experiments, the length of the embryos was significantly shorter in the *YWHAZ*(S230W)-expressing group. RNA doses are 2–4 ng.

To examine whether the *YWHAZ*(S230W) variant has altered activity from its wild type counterpart, we injected the RNAs encoding the wild type or the variant form of *YWHAZ* into the animal (ectodermal) or the marginal zone (mesodermal) regions of early *Xenopus* embryos. At the gastrula stages, many embryos injected with high doses of the S230W variant (2–4 ng) displayed dark pigmentation in the animal region, and the clusters of darkly pigmented cells persisted to the neurula stages (Figure 2A,B). This phenotype normally reflects changes in cell morphology in a process called apical constriction, which results in concentration of pigment granules near the apical cell surface of epithelial cells, hence the dark color of the cells on the surface (Sawyer et al., 2010). Although S230W might induce cell shape changes, it did not seem to induce definitive apical constriction as the epithelial cell sheet did not bend toward the pigmented cell clusters (data not shown). Embryos injected with wild type *YWHAZ* RNA only showed minor pigmentation changes (Figure 2A,B). At the tadpole stages, embryos injected with the *YWHAZ*(S230W) RNA in the marginal zone showed severe defects in the head structures and a shortened and bent body axis. In contrast, embryos expressing *YWHAZ* displayed much milder defects, with a lesser degree of head malformation and bending of the body axis (Figure 2C). The differential phenotypes induced by the same doses of RNAs of the wild type or the variant *YWHAZ* indicate that the variant has different activity from its wild type allele.

### YWHAZ(S230W) Rescues the Defects Induced by a Dominant Negative (DN) FGF Receptor More Efficiently Than YWHAZ

**FIGURE 3:**
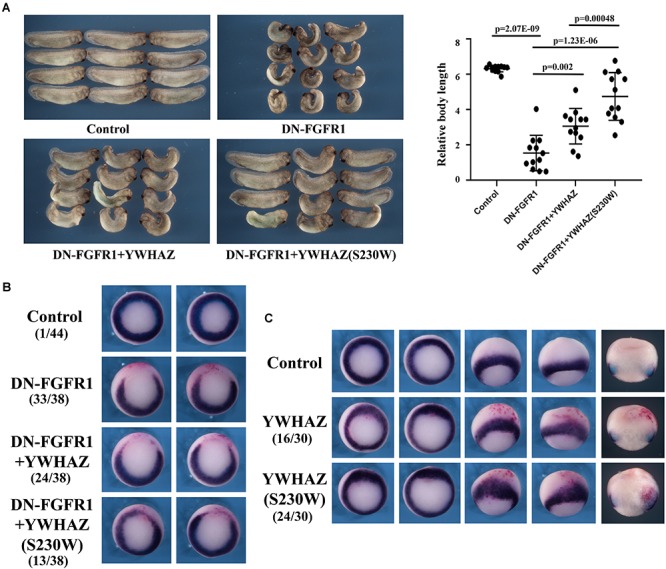
*YWHAZ*(S230W) rescues embryonic defects induced by DN-FGFR1 more efficiently than *YWHAZ*. **(A)** Expression of DN-FGFR1 (25 pg or 50 pg) in the dorsal marginal zone of early embryos results in gastrulation defects, characterized by reduced body length and exposed mesendoderm (“open back”). Co-expression of either *YWHAZ* or *YWHAZ*(S230W) (250 pg) with DN-FGFR1 leads to phenotypic rescue, but the S230W variant is more efficient in restoring body length and embryo morphology. The scatter plot of the body length of the embryos showed that the S230W variant rescued the defects more efficiently than YWHAZ. Student’s *t-tests* were performed in a pairwise fashion and the results revealed significant differences in the samples we compared. This experiment has been repeated four times, with 12 to 18 treated embryos in each experiment. Since the embryos were collected at slightly different stages and the body length was thus different, scatter plot was made for the embryos for each experiment separately. In all the experiments, there was a significant difference between *YWHAZ* and *YWHAZ*(S230W) in phenotype rescue. **(B)** DN-FGFR1 interferes with the expression of the pan-mesodermal marker brachyury, and *YWHAZ*(S230W) rescues marker expression to a greater extent compared to wild type *YWHAZ*. The experiment was repeated 3 times. For control embryos, 1/44 embryos showed a gap in the brachyury ring, and 33/38 embryos injected with *DN-FGFR1* RNA had a big gap in the brachyury domain. Expression of *YWHAZ* and *YWHAZ*(S230W) led to partial rescue of brachyury expression, resulting in similar brachyury gap in 24/38 and 13/38 embryos, respectively. These numbers are indicated in the panel. **(C)** Ectopic expression of *YWHAZ* weakly expands, whereas ectopic expression of *YWHAZ*(S230W) strongly expands, the domain of *brachyury* in gastrulating *Xenopus* embryos. The experiment has been repeated 3 times. The expansion of *brachyury* domain was observed in 16/30 embryos expressing *YWHAZ* and 24/30 embryos expressing *YWHAZ*(S230W). The doses of RNAs used in this panel are 1 to 2 ng. The red color in panels B and C are staining of beta-galactosidase-expressing cells with the chemical red-Gal to mark the injected cells.

Genetic variation identified in CFC individuals often shows gain-of-function activity, resulting in activation of the RAS-RAF-MEK-ERK pathway. If the S230W variant is responsible for the CFC phenotype in our proband, it is likely to promote Erk activation as well. To test this hypothesis, we examined the ability of *YWHAZ* or *YWHAZ*(S230W) to rescue the defects induced by blocking the FGF signal using dominant negative FGF receptor 1 (DN-FGFR1). In *Xenopus*, the FGF signal (e.g., bFGF/FGF2; Kimelman et al., 1988) activates the Raf-MEK-Erk pathway to regulate mesoderm formation (Amaya et al., 1991, 1993; MacNicol et al., 1993; LaBonne and Whitman, 1994; LaBonne et al., 1995; Umbhauer et al., 1995). Blocking the signal with DN-FGFR1 impairs mesoderm development, leading to gastrulation defects (Amaya et al., 1991, 1993). When we expressed the RNA encoding DN-FGFR1 in the dorsal marginal zone of early *Xenopus* embryos, we observed that the injected embryos frequently displayed a reduced and bent body axis, and some showed failure in blastopore closure (“open back” phenotype; Figure 3A). Co-expression of DN-FGFR1 with either *YWHAZ* or *YWHAZ*(S230W) led to rescue of embryo morphology, but the S230W variant was more efficient in its ability to restore axial structures (Figure 3A). *In situ* hybridization analysis of expression of the pan-mesodermal marker brachyury (Smith et al., 1991) showed that DN-FGFR1 interfered with expression of this marker, and *YWHAZ*(S230W) rescued marker expression to a greater extent than *YWHAZ* (Figure 3B). To investigate whether overexpression of *YWHAZ* or *YWHAZ*(S230W) was sufficient to induce the mesodermal marker in early embryos by itself, we co-injected RNAs encoding wild type *YWHAZ* or the S230W variant with the lineage tracer encoding nuclear beta-galactosidase into the marginal zone of early embryos. Detection of brachyury expression at mid-gastrula stages revealed that while neither gene product induced brachyury expression in the ectodermal region by itself, both could expand the expression domain of brachyury in the marginal zone. *YWHAZ* weakly enhanced the expression of the marker in the area labeled by the tracer (red color, Figure 3C), whereas *YWHAZ*(S230W) strongly expanded the width of the brachyury expression domain outside its normal territory (Figure 3C). Taken together, our data suggest that overexpression of either *YWHAZ* or *YWHAZ*(S230W) activates the mesodermal induction pathway downstream of the FGF signal in proper tissue contexts, and the S230W variant has higher activity than wild type *YWHAZ*. It is important to note that the activities of *YWHAZ*(S230W) were similar to that of the active mutant of MEK1, MEK1(SESE) (Gotoh et al., 1995). When expressed at high doses, MEK1(SESE) induced defects in body axis, even though it positively regulated mesodermal induction (Supplementary Figure 1A; Gotoh et al., 1995). The defect was likely due to the requirement of the proper levels of Erk signaling in gastrulation movements subsequent to mesodermal formation (Nie and Chang, 2007). When expressed at a lower dose, MEK1(SESE) rescued the defects induced by DN-FGFR1 in a similar way as *YWHAZ*(S230W) (Supplementary Figure 1B). The results imply that *YWHAZ*(S230W) activates Erk signaling in its rescue of the DN-FGFR1 phenotype.

### YWHAZ(S230W) Stimulates Raf-Dependent Erk Phosphorylation More Efficiently Than YWHAZ

**FIGURE 4:**
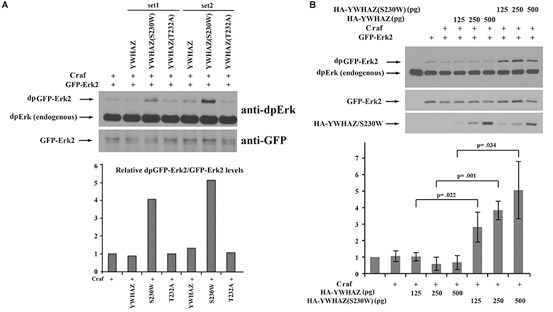
*YWHAZ*(S230W) strongly stimulates Raf-dependent Erk phosphorylation when compared with wild type *YWHAZ*. **(A)** Co-expression of Craf (250 pg) and GFP-Erk2 (250 pg) with wild type *YWHAZ*, the S230W variant, or the T232A mutant (250 pg) shows that only the S230W variant strongly enhances Craf-stimulated Erk phosphorylation. Quantification of the Western blot was performed using NIH ImageJ software and the relative ratio of phosphorylated GFP-Erk2 (dpGFP-Erks) over total GFP-Erk2 is shown in the bar graph. **(B)** Co-expression of Craf (125 pg), GFP-Erk2 (125 pg) and an increasing amount of HA-*YWHAZ* or HA-*YWHAZ*(S230W) (125, 250, and 500 pg) shows that the S230W variant stimulates Erk phosphorylation significantly more than *YWHAZ* at all doses used. The bottom bar graph displays the results of three independent experiments, with the average of relative Erk phosphorylation level (dpGFP-Erk2/total GFP-Erk2) and the standard deviation plotted on the graph. The *p-values* from Student’s *t-test* for each dose of *YWHAZ*/S230W are shown in the graph.

The above phenotypic and marker expression analyses imply that the S230W variant is more active than its wild type counterpart. To assess whether the functional difference between the two forms is based on their differential ability to activate the Erk pathway, we analyzed their effect on Erk phosphorylation. As *Xenopus* embryos have high endogenous Erk signals, especially in the mesodermal region (LaBonne and Whitman, 1997; Schohl and Fagotto, 2002), which can mask the effects of ectopically expressed *craf* and *YWHAZ*/S230W, we co-expressed these genes with GFP-Erk2 in the ectodermal region and assayed for phosphorylation of this chimeric protein. Ectodermal cells have low endogenous Erk signals, thus the level of GFP-Erk2 phosphorylation can reflect the activity of locally injected activator products. As shown in Figure 4A, *YWHAZ*(S230W), but not *YWHAZ*, strongly enhanced GFP-Erk2 phosphorylation when co-expressed with *craf*. Quantification of the ratio of phosphorylated over total GFP-Erk2 levels revealed that *YWHAZ*(S230W) stimulated Erk phosphorylation by 4 to 5 fold over that when *craf* was expressed alone, whereas wild type *YWHAZ* did not significantly change the ratio. This result was further confirmed by co-injection of a low dose of *craf* RNA with an increasing amount of RNAs of *YWHAZ* or *YWHAZ*(S230W), which demonstrated that the S230W variant had a statistically significant higher activity over that of *YWHAZ* in stimulating Erk phosphorylation (Figure 4B). To examine whether *YWHAZ* or *YWHAZ*(S230W) alone could induce Erk phosphorylation, we expressed these genes in the ectodermal region with GFP-Erk2 in the absence of *craf*. At higher doses (1 ng), *YWHAZ* and *YWHAZ*(S230W) weakly stimulated Erk phosphorylation, but the level of activation was lower compared to that when co-expressed with *craf* (compare data in Supplementary Figure 2 and Figure 4). The S230W variant again displayed higher activity than *YWHAZ* in this context (Supplementary Figure 2). These data indicate that like other CFC-associated variants, *YWHAZ*(S230W) functions as an activating mutant which facilitates Raf-Erk signaling.

### YWHAZ and YWHAZ(S230W) Do Not Promote Membrane Recruitment of Craf Protein

**FIGURE 5:**
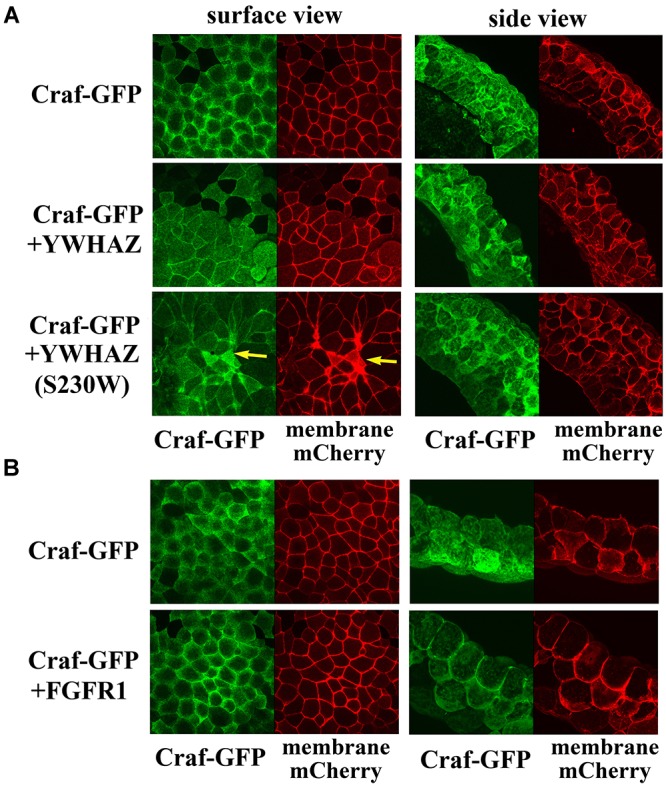
YWHAZ and YWHAZ(S230W) do not promote membrane recruitment of Raf. **(A)** Craf-GFP is localized both at the plasma membrane and in the cytosol in Xenopus ectodermal cells. Co-expression with YWHAZ or YWHAZ(S230W) does not significantly change the membrane fraction of the protein. The surface view reveals a population of Craf at the cell-cell junction in the presence of YWHAZ or the S230W variant in addition to the widespread Craf underneath the cell surface. Cell morphological changes are observed only in cells expressing the S230W variant and manifested as clusters of smaller cells displaying apical constriction-like morphology from the surface view (arrows). The side view shows intense Craf clusters at the juxtamembrane locations in cells expressing YWHAZ or YWHAZ(S230W). **(B)** Unlike wild type YWHAZ or its variant, FGFR1 recruits Craf to the plasma membrane in ectodermal cells. The doses of the RNAs used are: *Craf-GFP*, 250 pg, *YWHAZ*/*YWHAZ*(S230W), 2 ng, FGFR1, 1 ng.

Several distinct mechanisms may underlie the elevated activity of YWHAZ(S230W). YWHAZ normally binds to Raf proteins and holds them in a closed conformation in the cytosol. Upon stimulating signals to activate Ras, YWHAZ releases its binding at the N-terminal site of Raf proteins to allow their recruitment to the plasma membrane (Rommel et al., 1996; Tzivion et al., 1998; Light et al., 2002). It is possible that the S230W variant has reduced affinity for the N-terminal binding site of Raf to permit more efficient association of Raf with RAS and consequently better recruitment to the plasma membrane. To test this hypothesis, we made a Craf-GFP construct and examined its membrane localization in the presence of either YWHAZ or YWHAZ(S230W) by confocal microscopy. The RNA encoding a membrane-associated mCherry fluorescent protein was used to mark the plasma membrane in this experiment. Expression of Craf-GFP alone in the ectodermal cells resulted in both membrane and cytosolic localization of the protein (Figure 5A). The membrane association of Craf was likely due to the presence of endogenous FGF signal in these cells (Schohl and Fagotto, 2002). When Craf-GFP was co-expressed with either YWHAZ or YWHAZ(S230W), the Craf protein was seen from the surface view to spread underneath the cell membrane and at the cell-cell junctions. The widespread distribution under the plasma membrane did not seem to indicate a membrane recruitment of the protein, as the side view of the cells revealed a lack of membrane enrichment of the protein (Figure 5A). This pattern was distinct from that when Craf-GFP was co-expressed with FGFR1, in which case the Craf protein was detected to localize more intensely to the plasma membrane (Figure 5B). Although the effect of YWHAZ and YWHAZ(S230W) on Craf distribution seemed to be similar, we observed that YWHAZ(S230W)-expressing samples displayed some small cells with strong mCherry signal (yellow arrows, Figure 5A). This is consistent with our phenotypic studies at the gastrula stages when we observed darkly pigmented cells in the animal region (Figure 2A) and indicates that YWHAZ(S230W) could induce apical constriction-like cell shape changes. Taken together, our results show that YWHAZ and YWHAZ(S230W) do not promote membrane recruitment of Raf proteins, but YWHAZ(S230W) can induce cell morphological changes that are not obvious in YWHAZ-expressing samples.

### YWHAZ(S230W) Displays Increased Binding to Raf Proteins Compared With YWHAZ

**FIGURE 6:**
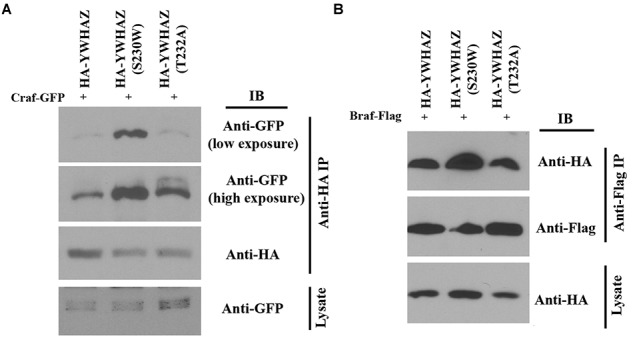
YWHAZ(S230W) binds to more Raf proteins than YWHAZ. Binding of HA-tagged YWHAZ(S230W) to either Craf (panel **A**) or Braf (panel **B**) is increased compared to that of HA-tagged YWHAZ. The doses of the RNAs used are: *Craf-GFP*, 250 pg, *Braf-Flag*, 125 pg, *HA-YWHAZ/HA-YWHAZ(S230W)/HA-YWHAZ(T232A)*, 250 pg.

Binding of YWHAZ to the Raf C-terminal phosphorylation site (S728 in BRAF and S621 in CRAF) is critical for Raf activation (Tzivion et al., 1998; MacNicol et al., 2000; Light et al., 2002; Garnett et al., 2005). The enhanced function of YWHAZ(S230W) in Raf-Erk activation may thus rely on differential ability of YWHAZ and YWHAZ(S230W) to interact with Raf. To explore this possibility, we performed co-immunoprecipitation (co-IP) studies to analyze the interaction of YWHAZ and the S230W variant with the Raf proteins. We co-injected RNAs encoding HA-tagged *YWHAZ*/S230W and *Craf-GFP* into the ectodermal region of early *Xenopus* embryos. Protein lysate was obtained at the gastrula stages and subjected to IP with anti-HA antibody. Western blot using anti-GFP antibody was then performed to examine the Craf-GFP protein that was pulled down by the anti-HA antibody. As shown in Figure 6A, the amount of Craf-GFP co-precipitated with HA-YWHAZ(S230W) was substantially higher than that pulled down by HA-YWHAZ, demonstrating that the S230W variant binds more Craf than wild type YWHAZ. Similarly, we performed co-IP with HA-YWHAZ/S230W and Braf-Flag and demonstrated that HA-YWHAZ(S230W) has an increased interaction with Braf-Flag than HA-YWHAZ (Figure 6B). These data thus reveal that the S230W variant differs from YWHAZ in its ability to bind to Craf and Braf.

### YWHAZ(S230W) Cannot Be Phosphorylated by Casein Kinase 1a

**FIGURE 7:**
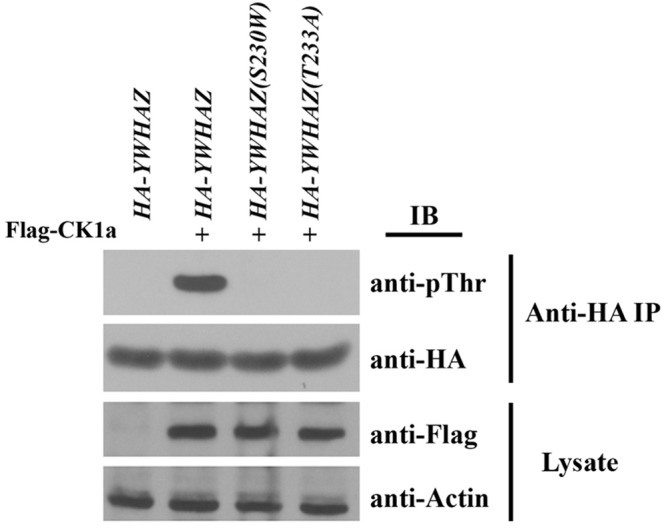
YWHAZ(S230W) cannot be phosphorylated by casein kinase 1a (CK1a). The S230W variant, like the phospho-null T232A mutant of YWHAZ, cannot be phosphorylated by CK1a. The doses of RNAs used are: *HA-YWHAZ/HA-YWHAZ(S230W)/HA-YWHAZ(T232A)*, 1 ng; *Flag-CK1a*, 500 pg.

It has been shown that binding of YWHAZ to Raf is affected by the phosphorylation state of YWHAZ itself. Casein kinase 1a (CK1a) can phosphorylate YWHAZ at threonine 232 (T232) to inhibit its association with CRAF (Rommel et al., 1996; Dubois et al., 1997; Obsilova et al., 2004). Since our variant alters the protein sequence at serine 230, which is positioned two residues away from T232, it is possible that the S230W variation will interfere with YWHAZ phosphorylation at T232, hence enhancing its interaction with Raf proteins. To investigate this hypothesis, we co-expressed RNAs of *HA-YWHAZ/HA-YWHAZ*(S230W) with that of *CK1a* and assessed phosphorylation of the protein by Western blot. In this experiment, we also used the YWHAZ(T232A) mutant that cannot be phosphorylated by CK1a as a control. As shown in Figure 7, CK1a phosphorylated YWHAZ efficiently, but was incapable of modifying either the T232A or the S230W mutant proteins.

To further investigate whether the loss of CK1a phosphorylation could account for the augmented activity of the S230W variant, we compared the functions of the S230W and the T232A proteins. Unlike the S230W variant, overexpression of the T232A mutant did not lead to elevated activation of Erk phosphorylation (Figure 4), did not enhance the binding to Raf over wild type YWHAZ (Figure 6), and did not rescue the embryonic defects induced by DN-FGFR1 (data not shown). These results imply that while loss of CK1a phosphorylation at Thr232 may impact binding of YWHAZ to Raf to some degree, it is not sufficient to activate Raf-Erk signaling. Additional factor(s) may influence the activity of the S230W variant.

## Discussion

With improved depth, precision, and reduced cost, genome or exome sequencing has been increasingly used to identify the molecular etiology of congenital diseases. As a result, a growing list of genomic variants have been uncovered which associate with specific pathological phenotypes. The pathogenicity of many of these variants, however, remains uncertain in the absence of supportive functional data. The bottleneck in assigning functional significance to variants is often the lack of assays to examine the activities of the variant gene products. Animal models, such as *Xenopus*, can be used to investigate gene function in an unbiased fashion. *Xenopus* embryos develop quickly, contain tissue types and organs equivalent to those in humans, undergo cell differentiation and morphogenesis both *in vivo* and in tissue explants, have conserved signaling and gene regulatory pathways, and are available for genetic, embryological, molecular, and biochemical studies. In this work, we have employed the *Xenopus* model to interrogate the function of a human gene variant that appeared to be associated with CFC syndrome.

Cardiofaciocutaneous syndrome is a rare autosomal dominant genetic disorder that affects 1 in 800,000 newborns (Roberts et al., 2006; Aoki et al., 2008; Dard et al., 2018). Currently, all known mutations linked to CFC syndrome act in the RAS-RAF-MEK pathway to enhance ERK signaling (Aoki et al., 2008; Simanshu et al., 2017; Bustelo et al., 2018; Dard et al., 2018). Although 14-3-3 proteins are thought to modulate RAF-ERK signaling both positively and negatively in mammalian cells, partially depending on the presence or absence of signals (Aitken, 2006; Morrison, 2008), no pathogenic variants in 14-3-3 family members have been identified in CFC patients. Genome sequencing of a CFC individual revealed a *de novo* heterozygous variant allele of the human 14-3-3ζ protein. If the variant is responsible for the CFC phenotype, it may act either as a haploinsufficient loss-of-function product which may impair Raf inhibition in cells or as a dominantly activating protein that promotes Erk signaling. Using the *Xenopus* overexpression system, we show here that the *YWHAZ*(S230W) variant induces more severe embryonic defects when ectopically expressed at high doses (2–4 ng) and rescues DN-FGFR1-induced defects more efficiently at low doses (0.25 ng) when compared with wild type *YWHAZ*. Analysis of mesodermal marker expression and Erk phosphorylation confirms that the variant enhances Erk signaling over that of *YWHAZ*. *YWHAZ*(S230W) expands the domain of the mesodermal gene brachyury more widely than its wild type counterpart; and the variant enhances the activation of Erk to higher levels than wild type *YWHAZ*. While definitive proof will require additional patients with variants in *YWHAZ*, our data, combined with the evidence that variations in other genes associated with CFC syndrome cause disease by stimulating Erk signaling, provide evidence that *YWHAZ* variation is causally related to CFC and developmental delay.

The mechanisms underlying the potential disease effects of the S230W variant is specifically investigated in this study. Unlike upstream growth factor signaling that activates Ras, such as the FGF pathway, neither YWHAZ nor its variant promotes membrane recruitment of the Craf protein (Figure 5). Instead, the S230W variant shows increased binding to both Braf and Craf when compared with wild type YWHAZ. Although phosphorylation of YWHAZ by casein kinase 1a is shown to inhibit the binding of YWHAZ to Raf in an *in vitro* assay using a Raf peptide substrate (Rommel et al., 1996; Dubois et al., 1997; Obsilova et al., 2004), and the S230W variant protein displays impaired phosphorylation by CK1a, loss of CK1a phosphorylation *per se* does not seem to contribute to the enhanced activity of the variant. The phospho-null mutant YWHAZ, YWHAZ(T232A), does not activate Erk signaling or rescue DN-FGFR1-induced defects any more than the wild type protein. Hence, the S230W variant seems to employ an alternative mechanism to bind Raf and activate Erk more efficiently. Inspection of the nature of the amino acid substitution in the context of the structural features of 14-3-3 proteins yields a clue to potential mechanisms. 14-3-3 proteins form dimeric, rigid cup shaped structures that contain a deep central channel with two amphipathic grooves that bind their substrates (Liu et al., 1995; Yaffe et al., 1997; Obsil and Obsilova, 2011). The *C-terminal* segment of 14-3-3 is located outside the cup-shaped structure and is more flexible. Nonetheless, this region can interact with both 14-3-3 itself and the partner proteins to regulate 14-3-3 conformation (Obsilova et al., 2004). The amino acid change in our YWHAZ variant occurs at position 230 to alter the serine residue into tryptophan. This residue is situated between the end of the helix that forms part of the ligand-binding groove of 14-3-3 and the beginning of the *C-terminal* stretch. Alteration of the amino acid sequence is thus expected to modify the conformation of the 14-3-3 dimer and influence its binding to partner proteins (Obsilova et al., 2004). We therefore speculate that structural changes in YWHAZ S230W variant can facilitate formation of a functional signaling complex by bringing downstream signaling components together more efficiently via the 14-3-3 dimer. This may involve promoting dimerization of BRAF and CRAF, stimulating formation of protein complexes between Raf and other signaling molecules, such as MEK or KSR, or shifting binding preference at the negative (e.g., S259 in CRAF) and the positive (e.g., S621 in CRAF) sites. Future biochemical studies combining Raf mutagenesis, for example *N*- and/or *C-terminal* serine to alanine (phospho-null) or aspartic acid (phosphomimetic), and protein interaction assays will help to shed further light on the mechanism of S230W action.

The structure of 14-3-3 proteins also provides some clues to the possible functional changes of the other YWHAZ variants we have identified. The amino acid alteration for G53R occurs in the helix 3, which is involved in both protein dimerization and ligand binding (Liu et al., 1995; Yaffe et al., 1997; Obsil and Obsilova, 2011). As glycine 53 normally induces a bend in this helix (Liu et al., 1995), the change would straighten the helix. Furthermore, the variant introduces a basic residue in the helix and may potentially strengthen the interaction with the phosphate group in the substrate (Yaffe et al., 1997). Hence the G53R variant may bind partners more strongly than the wild type protein. The S145L variant is in the helix 6, which is located outside the dimerization interface and the ligand binding groove. It is possible that the variant does not directly affect interactions with other proteins via its ligand binding channels, but may still alter the function of the protein by modification of auxiliary protein interactions at the outer surface. The S230Y-frameshift variant is interesting in that the first 229 amino acids, which form the rigid cup structure with the amphipathic ligand binding groove, are exactly the same as in the S230W variant, yet the two individuals with these variants show distinct phenotypes. This may be caused by the nature of the C-terminal amino acid sequence. In 14-3-3 proteins, the C-terminal region is flexible and can fold back to interact with the ligand binding groove in the absence of other partners (Liu et al., 1995). Removal of this region has been shown to enhance ligand binding by 14-3-3 proteins (Truong et al., 2002). The frameshift variation in S230Yfs^∗^44 alters the C-terminal sequence, which may either block ligand access more efficiently or enhance ligand binding. Alternatively, it is also possible that the frameshift leads to nonsense mediated decay of the messenger RNA of S230Yfs^∗^44 so that no protein will be made from the variant locus. Future functional, biochemical (protein stability, modification, and interaction) and structural analyses are required to determine the activities and the mechanisms of these other variants.

In *Xenopus*, as in other vertebrates, multiple 14-3-3 proteins play redundant roles in regulating early embryonic development (Wu and Muslin, 2002; Bunney et al., 2003; Muslin and Lau, 2005; Lau et al., 2006). Knockdown of individual 14-3-3 members results in either no defects, eye malformation, or impaired mesodermal induction and axial patterning defects (Lau et al., 2006). The results indicate that 14-3-3 proteins have overlapping and distinct functions in different tissue contexts. As 14-3-3 members can form both homo- and heterodimers, knockdown phenotypes likely do not capture the full range of 14-3-3 function during embryogenesis due to compensation. Tissue-specific overexpression or activation of 14-3-3 proteins have not been carried out, hence it is unclear whether the spectrum of morphological changes seen in CFC syndrome can be faithfully recapitulated if 14-3-3ζ is activated from its endogenous locus or replaced with a S230W equivalent allele in *Xenopus*. Nevertheless, our studies demonstrate that the *Xenopus* model can be used effectively to determine functional consequences of human genetic variants. This type of study may help to pave the way to use *Xenopus* embryos to screen for drugs that can modulate the activities of gene variants in the future.

## Materials and Methods

### Identification of YWHAZ Variants Using Genomic Medicine Approaches

For proband 1, trio whole genome sequencing and variant calling were performed as previously described (Bowling et al., 2017). For proband 2, trio whole exome sequencing was performed on a NextSeq 500 Sequencing System (Illumina, San Diego, CA, United States), with a 2 × 150 bp high output sequencing kit after a 12-plex enrichment with SeqCap EZ MedExomekit (Roche, Basel, Switzerland), according to manufacturer’s specifications. Sequence quality was assessed with FastQC 0.11.5, then the reads were mapped using BWA-MEM (version 0.7.13), sorted and indexed in a bam file (samtools 1.4.1), duplicates were flagged (sambamba 0.6.6), coverage was calculated (picard-tools 2.10.10). Variant calling was done with GATK 3.7 Haplotype Caller. Variants were then annotated with SnpEff 4.3, dbNSFP 2.9.3, gnomAD, ClinVar, HGMD, Variome Great Middle East and an internal database. Coverage for these samples was 93% at a 20x depth threshold. For probands 3, 4 and 5, exome sequencing was outsourced to GenomeScan (Leiden, The Netherlands). In brief, exomes were captured using the Agilent SureSelectXT Human all Exon v5 library kit (Agilent, Santa Clara, United States) accompanied by Illumina paired end sequencing on the Hiseq2500 platform (Illumina, San Diego, United States), generating 2 × 150 bp paired end reads with at least 80 × median coverage. Data analysis was performed at the LDGA using the in-house sequence analysis pipeline “Modular GATK-Based Variant Calling Pipeline” (MAGPIE) (LUMC Sequencing Analysis Support Core, LUMC) based on read alignment using Burrows-Wheeler Alignment (BWA-MEM) and variant calling using the Genome Analysis Toolkit (GATK). LOVDplus (Leiden Genome Technology Center, LUMC, Leiden) was used for interpretation of variants. Variants were classified according to the American College of Medical Genetics and Genomics (ACMG) guidelines. Written informed consent to publish pictures was obtained from all families.

### Obtaining Embryos and Microinjection

*Xenopus laevis* frogs were used in this study according to the institutional IACUC protocol 09658. Female frogs were primed with 800 units/frog of human chorionic gonadotropin hormone (Sigma) the night before usage. Embryos were obtained by *in vitro* fertilization, dejellied with 2% cysteine solution, and micro-injected with the RNAs indicated in the text. The injection was done in the animal regions of both blastomeres of 2–cell stage embryos or the marginal zone regions of the two dorsal cells of 4-cell stage embryos. The doses of the RNAs used in each experiment were indicated in the figure legends.

### Plasmid Constructs and RNA Synthesis

The construct containing the human YWHAZ sequence, pMCSG19-YWHAZ, was purchased from the Plasmid Repository at Arizona State University^1^ and was cloned into the pCS105 vector. The Craf, Braf and CK1a sequences were isolated by PCR amplification from *Xenopus* cDNA using the sequence reference from Xenbase^2^ (RRID:SCR_003280; James-Zorn et al., 2015; Karimi et al., 2018) and cloned into pCS105. The protein tags were added and the mutagenesis of YWHAZ was performed using the PCR-based method. All constructs were sequenced to confirm their identity. The plasmids were linearized with the AscI enzyme and transcribed using the SP6 mMessage mMachine RNA synthesis kit (Ambion) to make RNAs for injection.

### *In situ* Hybridization (ISH)

*In situ* Hybridization was performed as described by Harland (1991).

### Imaging and Embryo Quantification

For stereo imaging of embryonic phenotypes and *in situ* hybridization, Nikon AZ100 microscope was used. The body length of the embryos was measured as a straight line from the head to the tip of the tail, which would reflect the effects of both the length and the curvature. NIH ImageJ software was used in body length measurement, and the scatter plot was made using GraphPad Prism 8 software. Student’s *t-test* was used to determine statistical significance of the changes in body length of different samples in pairwise comparisons. For confocal imaging of Craf-GFP localization, Olympus Fluoview 2000 upright confocal microscope was used. Most of the images were taken using a 20× (NA0.95) lens. Maximum intensity projections of Z-stack images were used for the figures.

### Western Blot

Western blot was performed as described previously (Tien et al., 2015). Briefly, RNAs encoding tagged proteins were injected into 2- to 4-cell stage embryos. Embryonic lysate was obtained at gastrula stages by lysing embryos with cold buffer containing 50 mM Tris–HCl, pH 7.5, 150 mM NaCl, 1mM EDTA, 10% glycerol and 0.5% Triton X-100. The lysate was either loaded on the SDS–PAGE directly (for the dpErk assay) with the 2× SDS gel-loading buffer (100 mM Tris.Cl, pH6.8, 200mM DTT, 4% SDS, 0.2% bromophenol blue, 20% glycerol) or subjected to the co-IP assay. Western blot was performed using rabbit anti-Flag (1:3000, Sigma), rabbit anti-HA (1:3000, Cell Signaling Technology), rabbit anti-dpErk (1:4000, Cell Signaling Technology), rabbit anti-GFP (1:3000, Cell Signaling Technology), or mouse anti-p-threonine (1:2000, Cell Signaling Technology) antibodies. Quantification was performed using NIH ImageJ software. The Student’s *t-test* was used to assess the statistical significance in differences between YWHAZ and YWHAZ(S230W) in dose-response experiments shown in Figure 4.

## Ethics Statement

This study was carried out in accordance with the recommendations of “the UAB Institutional Review Board for Human Use (IRB)” with written informed consent from all subjects. The protocol was approved by the “The UAB Institutional Review Board for Human Use (IRB).” This study was carried out in accordance with the recommendations of “UAB IACUC committee.” The protocol was approved by the “UAB IACUC.” All subjects gave written informed consent in accordance with the Declaration of Helsinki.

## Author Contributions

SW, AvH, and BRK were involved in patient recruitment, examination, and clinical diagnosis. SH, BK, CR, GC, and M-JC performed genomic analyses. IP and CC carried out Xenopus studies to characterize the wild type and the variant gene products.

## Conflict of Interest Statement

The authors declare that the research was conducted in the absence of any commercial or financial relationships that could be construed as a potential conflict of interest.

## Footnotes

1 https://dnasu.org/DNASU/

2 http://www.xenbase.org/

## Supplementary Material

FIGURE S1 Activated MEK1 induces gastrulation defects when expressed at high doses and rescues defects induced by DN-FGFR1 when expressed at low doses. **(A)** Injection of 0.5 ng RNA of MEK1(SESE), an activated MEK1 mutant, induces gastrulation defects. **(B)** When expressed at a lower dose (0.1 ng), MEK1(SESE) rescues defects induced by DN-FGFR1 to a similar degree to that when *YWHAZ*(S230W) was used, and both were more efficient than *YWHAZ*. This experiment has been repeated twice and the results from both experiments are similar.

FIGURE S2 High doses of *YWHAZ* and *YWHAZ*(S230W) weakly stimulate Erk phosphorylation. When injected at the 1 ng doses into the ectodermal cells, *YWHAZ* and *YWHAZ*(S230W) activate Erk signaling weakly. The level of activation is much lower than that when these genes were co-expressed with Raf, as seen in Figure 4.

TABLE S1 Genotypes and phenotypes of individuals with variation in YWHAZ.
